# Physical Therapy Management of Scapular Dyskinesis Due to Spinal Accessory Nerve Palsy Secondary to Neuralgic Amyotrophy: A Case Report

**DOI:** 10.7759/cureus.104935

**Published:** 2026-03-09

**Authors:** Michael P Groesch

**Affiliations:** 1 Physical Therapy, Cleveland Clinic, Mayfield Heights, USA

**Keywords:** brachial neuritis, manual therapy, neuralgic amyotrophy, parsonage-turner syndrome, physical therapy, scapular dyskinesis, spinal accessory nerve palsy

## Abstract

This case report describes a rare clinical presentation of spinal accessory nerve (SAN) palsy secondary to neuralgic amyotrophy and discusses physical therapy differential diagnosis, interventions, and outcomes. The patient was a 37-year-old left-hand-dominant Caucasian male who presented to physical therapy with right-sided neck and shoulder pain and limited right shoulder overhead movement. Examination identified trapezius atrophy, weakness, compensatory scapular protraction during shoulder flexion, and a positive modified Scapular Assistance Test. Physical therapy interventions included a multimodal approach with therapeutic exercise focused on scapular stabilization and shoulder strengthening, as well as manual therapy interventions including dry needling, thoracic manipulation, elastic therapeutic taping, scapular stretching, and mobilizations with movement to reduce pain and facilitate improved trapezius activation. The patient was seen for ten sessions over four months, with significant improvement in right shoulder flexion active range of motion from 125° to 155° within four weeks, ultimately achieving 165°. He also demonstrated improvements in shoulder strength but continued to exhibit trapezius weakness. Final outcome measures were mixed, with improvement on the Global Rating of Change (GRC) scale by +5, indicating “quite a bit better” and meeting the threshold for meaningful improvement, and improvement on the Patient-Reported Outcomes Measurement Information System (PROMIS) self-efficacy subscale by 4 points, from 37 to 41; however, his PROMIS physical function score declined from 46 to 31. Physical therapists should be aware of neuralgic amyotrophy affecting the SAN as a rare cause of scapular dyskinesis. Early conservative management with physical therapy using a multimodal approach, including manual therapy, was associated with rapid restoration of shoulder flexion range of motion and improvements in strength and pain management in this case.

## Introduction

Neuralgic amyotrophy, also known as Parsonage-Turner syndrome or brachial neuritis, is a painful peripheral axonal neuropathy that can be triggered by mechanical stress, viral infection, vaccination, pregnancy, or surgery [1,2]. This condition is thought to have an inflammatory autoimmune pathophysiology that occurs following a triggering event and presents with a rapid onset of severe pain followed by amyotrophy, or progressive wasting of the involved muscles [1-4]. Neuralgic amyotrophy occurs more often in men and most commonly affects the long thoracic and suprascapular nerves, although it can also involve nerves from the cervical plexus, lumbosacral plexus, and cranial nerves [1,4,5]. Recovery is typically slow and can take months to years for a partial to complete return of nerve function [2,3,6].

Neuralgic amyotrophy affecting the spinal accessory nerve (SAN) is rare, but isolated cases have been reported [1,2,4]. This condition is a diagnosis of exclusion, making it difficult to differentiate from a compression palsy and often leading to underdiagnosis when the SAN is involved [1,2,7]. Injury to the SAN can cause weakness and atrophy in both the sternocleidomastoid and trapezius muscles, depending on the site of injury along the nerve [8,9]. The SAN pathway begins with two contributions: a cranial portion from the nucleus ambiguus of the medulla and a cervical portion from the anterior horn cells of the upper four cervical vertebrae [8,9]. These portions merge into the SAN before it exits the jugular foramen of the skull [8,9]. The nerve then branches to innervate the sternocleidomastoid before continuing through the posterior triangle of the neck, where it is superficial and most susceptible to injury [8,9]. Because of this branching pattern, injuries in the posterior triangle may not affect sternocleidomastoid function, a distinction that can help localize the site of SAN injury [7,8].

Evidence on effective physical therapy interventions for neuralgic amyotrophy is limited. Reported interventions include scapular stabilization, motor control and coordination exercises, shoulder range-of-motion exercises, neurodynamic techniques, and progressive shoulder strengthening during later rehabilitation phases [2,3,6,10-12]. Manual therapy interventions have also been described, including scapular mobilization, elastic therapeutic taping, thoracic mobilization, dry needling, mobilizations with movement (MWM), and soft tissue mobilization [2,10-12].

This case report describes a rare clinical presentation of SAN palsy (SANP) secondary to neuralgic amyotrophy triggered by mechanical stress and discusses physical therapy differential diagnosis, interventions, and outcomes. To the author’s knowledge, this is the first case report on physical therapy intervention for neuralgic amyotrophy isolated to the SAN.

## Case presentation

The patient was a 37-year-old left-hand-dominant Caucasian male referred to physical therapy with a diagnosis of chronic right shoulder pain, scapular dyskinesis, and right rotator cuff dysfunction. He presented with pain on the right side of his neck and shoulder, which began approximately three months prior to evaluation, and he reported difficulty with overhead reaching. The patient first noticed symptoms the day after heavy weightlifting that included upright rowing. He also reported that part of his exercise routine involved walking long distances while wearing a weighted vest.

The patient’s past medical history included depression, and he was not taking any medications at the time of the initial evaluation. His goal was to reduce pain and return to work, which required regularly lifting up to 50 lbs. His pain was located at the right superior angle of the scapula and was described as dull and aching, with intensity rated 2/10 at rest and 10/10 at worst. He also reported numbness and tingling in his right upper extremity when symptoms first began; these symptoms now occurred only intermittently with upper extremity movements, without a consistent pattern.

Following the subjective examination, potential diagnoses included scapular dyskinesis, rotator cuff dysfunction, and cervical radiculopathy. Cervical radiculopathy was considered due to intermittent radicular symptoms into the right upper extremity, while rotator cuff dysfunction and scapular dyskinesis were considered due to pain and difficulty with overhead reaching.

Examination

The initial examination identified limited active right shoulder flexion and functional external rotation without pain (Table 1). Notably, when attempting overhead reaching, the patient demonstrated significant protraction of the right scapula, with shoulder flexion limited to 125°. During the modified Scapular Assistance Test (SAT), he achieved full pain-free right shoulder flexion, confirming the diagnosis of scapular dyskinesis [13].

**Table 1:** Initial and final active range of motion measurements (degrees)

Shoulder movement	Left		Right	
	Initial	Final	Initial	Final
Shoulder flexion	160	160	125	165
Shoulder abduction	-	150	-	145
Shoulder functional internal rotation	T12	T12	T12	T10
Shoulder functional external rotation	T4	T4	C7	T3

Palpation reproduced concordant pain at the right superior angle of the scapula, levator scapulae, and upper trapezius. The resting scapular position was protracted with slight medial rotation, and winging of the medial border that caused the superior angle of the scapula to slightly protrude. Manual muscle testing revealed strength impairments in the right middle trapezius (3+/5), lower trapezius (2+/5), shoulder external rotation (4+/5), flexion (4/5), and abduction (4/5), with pain reproduced during testing of shoulder external rotation, flexion, and abduction.

Neurological examination included dermatome, myotome, and reflex testing due to the patient’s report of intermittent symptoms radiating into the right upper extremity. Findings indicated a reduced right biceps reflex (1+) and weakness in the right C5 myotome. Other upper extremity reflexes assessed, such as bilateral brachioradialis, triceps, and left biceps, were all 2+. Sensation testing showed no changes bilaterally. Cervical spine provocation testing, including Spurling’s A, was negative, and there was no reproduction of right upper extremity radicular symptoms throughout the examination. The inability to reproduce upper extremity symptoms with provocation testing, normal right cervical rotation range of motion, and normal sensation findings made cervical radiculopathy less likely.

The patient also completed the Patient-Reported Outcomes Measurement Information System (PROMIS), scoring 46 on the physical function subscale, indicating normal perceived ability to perform activities of daily living and physical tasks, and 37 on the self-efficacy subscale, indicating low confidence in his ability to manage symptoms [14].

Diagnosis and prognosis

The initial examination supported a diagnosis of scapular dyskinesis due to compensatory scapular protraction during overhead movement, improvement in shoulder motion during the modified SAT, and weakness on manual muscle testing of the right middle and lower trapezius (Table 2).

**Table 2:** Differential diagnosis Summary of relevant impairments from the examination for each diagnosis. While some findings suggested a contribution to symptoms from cervical radiculopathy or rotator cuff dysfunction, the examination findings most strongly supported a diagnosis of scapular dyskinesis. Normal cervical rotation range of motion to the ipsilateral side, normal sensory testing, and no reproduction of upper extremity symptoms with Spurling’s A made cervical radiculopathy less likely. Neither cervical radiculopathy nor rotator cuff dysfunction would account for the scapular dyskinesis identified during the examination. SAT, Scapular Assistance Test

Scapular dyskinesis	Cervical radiculopathy	Rotator cuff dysfunction
Significant improvement in motion during the modified SAT, weakness in the middle trapezius (3+/5) and lower trapezius (2+/5)	Reduction in right biceps reflex (1+), C5 myotome strength deficits, no reproduction of radicular symptoms, negative Spurling’s A, no limitation in right cervical rotation	Pain and weakness in right shoulder external rotation (4+/5)

The initial prognosis for therapy was favorable due to involvement of his non-dominant upper extremity, his overall good health status, and initial within-session improvements [15]. The initial treatment plan consisted of one visit per week for four visits using a multimodal approach of therapeutic exercise and manual therapy. Therapeutic exercise interventions focused on scapular stabilization, motor control, and shoulder strengthening, while manual therapy interventions addressed pain and facilitated improved lower and middle trapezius activation.

At the second visit, observation of the trapezius revealed atrophy compared to the contralateral side. Further neurological screening identified impaired SAN function, with asymmetrical weakness in the right sternocleidomastoid (3+/5) and upper trapezius (3+/5). Reflexes were reassessed, showing improvement of the right biceps reflex to 2+, and upper motor neuron reflexes, including Hoffman’s sign and the inverted supinator sign, were both absent. Cranial nerve assessment revealed no additional abnormalities. The referring provider was contacted regarding concern for SAN injury, and additional diagnostic testing was ordered.

The patient completed diagnostic testing concurrently with physical therapy, including cervical radiography, cervical MRI, and electrodiagnostic testing with both nerve conduction studies and needle electromyography. Cervical spine radiographs demonstrated degenerative changes of the atlantoaxial joint but no evidence of dynamic instability. Cervical MRI showed degenerative changes, most prominent at C5-6 and C6-7. Electrodiagnostic testing revealed reduced right SAN motor response compared to the left side and axonal loss changes in the right trapezius and sternocleidomastoid with active denervation (Table 3). These results confirmed a moderate right SAN mononeuropathy, and the patient was diagnosed with SANP; the underlying cause remained unknown.

**Table 3:** Electrodiagnostic testing ADM, abductor digiti minimi; Amp, amplitude; APB, abductor pollicis brevis; cervical PSP (high), cervical posterior root sensory potential; CV, conduction velocity; Dur, duration; EMG, electromyography; F-wave, late motor response; Fasc, fasciculations; Fib, fibrillation potentials; Ins Act, insertional activity; LatNpk, latency of the negative peak; LatOn (ms), latency onset; NC, normal characteristics; NE, no evidence; NRLX, not relaxed; Poly, polyphasic; PW, positive wave; R/L, right/left; Recruit, motor unit recruitment pattern; SCM, sternocleidomastoid; Temp, skin temperature

Sensory nerve conduction																	
Nerve	Stimulus	Recording	B-P Amp L (uV)	B-P Amp R (uV)	LatNpk L (ms)	LatNpk R (ms)	CV L (m/s)	CV R (m/s)	Dist L (mm)	Dist R (mm)	Norm B-P Amp	Norm LatNpk	Norm CV	Temp L (C)	Temp R (C)		
Median	Wrist	Index		49.84		3.24				130	>20 uV	<3.4 ms	>50 m/s		31.3		
Ulnar	Wrist	Fifth digit		37.48		2.9				110	>12 uV	<3.1 ms	>50 m/s		31.3		
Radial	Thumb	Forearm		44.79		2.26				100	>18 uV	<2.7 ms			31.3		
Motor nerve conduction																	
Nerve	Recording	Stimulus	B-P Amp L (mV)	B-P Amp R (mV)	LatOn L (ms)	LatOn R (ms)	CV L (m/s)	CV R (m/s)	Dist L (mm)	Dist R (mm)	Norm B-P Amp	Norm LatOn	Norm CV	Temp L (C)	Temp R (C)		
Median	APB	Wrist		7.71		3.8				50	>6 mV	<3.9 ms	>50 m/s		31.5		
Median	APB	Elbow		6.33		8		59.5		250					31.4		
Ulnar/ADM	ADM	Wrist		11.18		2.6				50	>7 mV	<3.1 ms	>50 m/s		31.2		
Ulnar/ADM	ADM	Below elbow		8.94		5.75		66.7		210					30.9		
Ulnar/ADM	ADM	Above elbow		9.12		7.7		60.8		310					31.1		
Spinal accessory	Trapezius	SCM	10.21	5.14	1.8	1.95											
F-wave side-to-side comparison																	
Nerve	Stimulus	Recording	F-Wave Lat L (ms)	F-Wave Lat R (ms)													
Ulnar/ADM	Wrist	ADM		28.75													
Needle EMG summary																	
Side	Muscle	Ins Act.	Fib	PW	Fasc	Other	Number	Recruit	Dur	Dur	Amp	Amp	Poly	Poly	Descript	Descript	Descript
L	Trapezius	Norm	0	0	0		1-	Mod		Norm		Norm		Norm	NC	NC	NC
	SCM						Norm	Full		Norm		Norm		Norm	NRLX	NC	NC
R	First dorsal inter	Norm	0	0	0		Norm	Full		Norm		Norm		Norm	NC	NC	NC
	Flex. pollicis longus	Norm	0	0	0		1-	Mod		Norm		Norm		Norm	NC	NC	NC
	Extn. indicis pro	Norm	0	0	0		Norm	Full		Norm		Norm		Norm	NC	NC	NC
	Pronator teres	Norm	0	0	0		2-	Mod-V		Norm		Norm		Norm	NC	NC	NC
	Biceps brachii	Norm	0	0	0		Norm	Full		Norm		Norm		Norm	NC	NC	NC
	Triceps - lat	Norm	0	0	0		1-	Mod		Norm		Norm		Norm	NC	NC	NC
	Deltoid, middle	Norm	0	0	0		Norm	Full		Norm		Norm		Norm	NC	NC	NC
	Rhomboid major	Norm	0	0	0		Norm	Full		Norm		Norm		Norm	NC	NC	NC
	Trapezius (upper)	Norm	2+				3-	Mod-V	Some	1+		Norm	Some	1+	NC	NC	NC
	SCM	Norm					2-	Mod-R	Some	1+	Some	1+	Some	1+	NRLX	NC	NC
	Cervical PSP (high)	Norm	0	0	0			NE							NC	NC	NC

Following consultations with neurology, additional diagnostic imaging was ordered, including a CT scan of the cervical spine and MRI of the brain, to rule out a proximal lesion as the cause of the SAN injury. The cervical CT scan demonstrated degenerative changes without fracture, and the brain MRI was unremarkable. After multiple consultations with neurology and neurosurgery, the cause of the SANP was suspected to be neuralgic amyotrophy, based on the patient’s history, findings from electrodiagnostic testing, and negative results on imaging studies of the cervical spine and brain. The time from symptom onset to final diagnosis was nine months, approximately three months after formal physical therapy had been completed.

Intervention

Interventions included a multimodal approach with therapeutic exercise focused on scapular stabilization, motor control, and shoulder strengthening, along with manual therapy interventions to address pain and facilitate improved trapezius activation. Manual therapy was initiated at the initial evaluation with a prone mid-thoracic grade 5 thrust manipulation and manual stretching of the scapula into depression, retraction, and posterior tilting. Therapeutic exercises began with thoracic and pectoralis minor flexibility, scapular stabilization exercises, and shoulder strengthening while maintaining the shoulder below 90° (Table 4). The patient demonstrated a positive response to initial interventions, with an intrasession improvement in active right shoulder flexion from 125° to 135°.

**Table 4:** Phase one exercise intervention An asterisk (^*^) indicates that the exercise was included in the home exercise program.

Exercise	Resistance	Sets	Repetitions	Hold time (seconds)
Pectoralis minor doorway stretch^*^		1	4	30
Scapular retractions^*^		2	10	5
Shoulder extension^*^	Extra heavy resistance band	2	10	
Rows^*^	Extra heavy resistance band	2	10	
Serratus punch^*^	Extra heavy resistance band	2	10	
Shoulder external rotation^*^	Medium resistance band	2	10	
Thoracic extension on chair^*^		1	10	5
Scaption to 90°^*^	2-3 lbs	2	10	
Prone horizontal abduction^*^	2-3 lbs	2	10	
Prone shoulder extension^*^	2 lbs	2	10	
Shoulder circles the ball on the wall		2	20	

**Figure 1:**
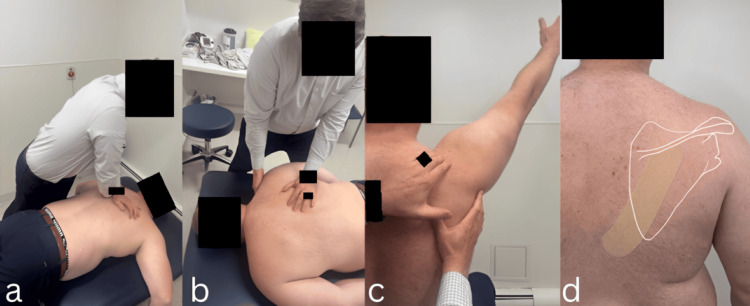
Manual therapy techniques Demonstration of manual therapy techniques used to address pain, facilitate improved trapezius activation, and improve shoulder flexion range of motion: (a) Prone mid-thoracic manipulation; (b) Prone manual stretching of scapula into posterior tilting; (c) Mulligan Mobilization with Movement into right shoulder flexion; (d) Elastic therapeutic taping application from mid-scapula across the lower border of the scapula toward midline.

Manual therapy interventions continued over the next three visits, with the addition of Mulligan MWM into right shoulder flexion and abduction, dry needling to the right upper trapezius and semispinalis capitis, and application of elastic therapeutic tape across the inferior border of the scapula to facilitate improved lower trapezius function (Figure 1). Dry needling was performed in the prone position on the right upper trapezius using two 0.30 × 30 mm needles inserted at superior medial and lateral angles into trigger points and on the semispinalis capitis using two 0.30 × 30 mm needles inserted lateral to the spinous process and angled 45° medially and 5° inferior toward the lamina. The patient continued to have a favorable response to these manual therapy interventions, with intrasession improvements in shoulder flexion active range of motion of 10-20° at each visit, along with short-term pain relief. Scapular stabilization and right shoulder strengthening exercises were progressed over these visits with increasing resistance.

By the fourth visit, the patient demonstrated 155° of right shoulder flexion, along with improvements in right shoulder external rotation strength to 5/5 and horizontal abduction to 4+/5. He continued to demonstrate asymmetrical weakness in his right upper, middle, and lower trapezius muscles. The patient reported an overall 80-90% improvement but still experienced daily intermittent pain on the right side of his neck, rated 7/10 at worst. At this time, the patient was undergoing additional testing concurrent with physical therapy for suspected SANP, and the frequency of interventions was reduced to one visit every two weeks, as the anticipated timeframe for return of trapezius strength had increased.

Over the remaining visits, the patient continued to improve right shoulder flexion active range of motion to 165° but demonstrated an inability to maintain this overhead position. Treatment advanced to phase two, with decreased focus on manual therapy and increased focus on progression of therapeutic exercise, including upper trapezius and overhead strength and endurance training (Table 5). Serratus anterior strength was also assessed on the right upper extremity during this phase, with a manual muscle testing grade of 4+/5 on the involved extremity and 5/5 on the uninvolved extremity.

**Table 5:** Phase two exercise progressions An asterisk (^*^) indicates additions to the home exercise program during phase two interventions.

Exercise	Resistance	Sets	Repetitions
Wall slide		2	10
Shoulder flexion serratus activation^*^	Light resistance band	2	10
Standing overhead ball pass on the wall		2	20
Lower trapezius setting at the wall^*^		3	10
Standing overhead press against the wall		3	10
Proprioceptive neuromuscular facilitation D2 flexion (supine to standing)	Light resistance band	2	10
Side-lying shoulder abduction		3	10
Shoulder shrugs^*^	10 lbs	2	10

Manual therapy interventions during this phase included prone mid-thoracic manipulations and manual scapular stretching to address the patient’s persistent right-sided neck and scapular pain, along with MWM to improve scapular motor control during overhead movements (Table 6).

**Table 6:** Manual therapy interventions performed during each visit MWM, mobilizations with movement

Manual therapy technique	Phase 1 (visits 1-4)	Phase 2 (visits 5-10)
Mid-thoracic manipulation	1-4	5-6
Manual scapula stretching	1-4	5-6
Mulligan MWM	2-4	8-9
Dry needling	2-4	-
Elastic therapeutic tape	2-4	-

Outcomes

The patient was seen in physical therapy for a total of 10 visits over four months. Overall, he improved his right shoulder flexion active range of motion to 165°, with an improved ability to maintain his upper extremity overhead. He also demonstrated improved right shoulder strength; however, he continued to have asymmetrical weakness in the right upper, middle, and lower trapezius (Table 7). He continued to experience moderate levels of pain, rated 5/10 at worst after physical activity, which improved from 10/10 at worst at the initial evaluation. Subjectively, he reported 70% overall improvement and rated his progress on the Global Rating of Change (GRC) as +5, indicating “Quite a bit better” and meeting the level for clinically meaningful improvement [16]. His PROMIS self-efficacy score improved by 4 points, from 37 to 41; however, his PROMIS physical function score declined from 46 to 31, indicating moderate dysfunction in his perceived ability to perform physical activities of daily living and physical tasks.

**Table 7:** Initial and final strength measurements (manual muscle testing)

Assessed muscle/movement	Left		Right	
	Initial	Final	Initial	Final
Shoulder internal rotation	5/5	5/5	5/5	5/5
Shoulder external rotation	5/5	5/5	4+/5 (painful)	4+/5
Horizontal abduction	4/5	4/5	4-/5	4+/5
Lower trapezius	4-/5	4+/5	2+/5	2+/5
Middle trapezius	5/5	5/5	3+/5	4-/5
Upper trapezius	5/5	5/5	3+/5	4/5

The patient did not achieve his goal of returning to full-duty work, as he was only able to lift 21 lbs on a functional capacity evaluation, whereas his job required regularly lifting 50 lbs. The continued strength limitations were expected, as full recovery of nerve function from neuralgic amyotrophy can take years. The patient chose at this time to continue with the prescribed exercises independently.

## Discussion

This case demonstrates a rare presentation of scapular dyskinesis due to SANP secondary to neuralgic amyotrophy in a patient presenting for physical therapy evaluation. SAN injuries and neuralgic amyotrophy can often go undiagnosed or misdiagnosed for an extended period, with the final diagnosis in this case occurring nine months after symptom onset [1,2,4,9]. Neuralgic amyotrophy can be difficult to distinguish from a compression palsy, as it is a diagnosis of exclusion, but several characteristics in this case favor it as the likely diagnosis: the presence of a mechanical stress trigger preceding the onset of pain, followed by muscle atrophy, and involvement of both the sternocleidomastoid and trapezius on electrodiagnostic testing in the absence of a proximal lesion on imaging studies [1,2,7]. The trigger in this case was believed to be mechanical stress due to the patient’s history of weightlifting the day before symptom onset.

The physical therapist was the first clinician to suspect SAN injury in this case, demonstrating the importance of recognizing this presentation for physical therapists, especially those practicing in direct-access roles. The clinical features that led to early suspicion of SANP included atrophy of the right upper trapezius, asymmetrical weakness of the right trapezius and sternocleidomastoid, and limited right shoulder active range of motion with compensatory scapular protraction during forward flexion. The increased protraction observed during overhead arm movement was likely due to the patient compensating with increased reliance on the serratus anterior to perform upward rotation of the scapula because of impaired trapezius function. Recognition of this compensatory protraction may be an important observational sign to detect SAN impairment. The patient’s limited shoulder flexion active range of motion also improved to full motion during the modified SAT, which has been shown to have adequate reliability for identifying the scapula as a contributor to shoulder pain and dysfunction [13]. Clinicians should have increased suspicion for SAN injury if either of these signs is present during examination.

The findings in this case add to other observable signs used to identify SANP, including the scapular flip sign, the triangle sign, and the active elevation lag sign. The scapular flip sign is positive if the medial border of the scapula lifts from the thoracic wall during resisted shoulder external rotation, indicating a loss of medial scapular stability [9]. The triangle sign is present when there is compensatory spinal hyperextension during forward elevation of the arm in prone, and the active elevation lag sign is present when there is an observable lag between arms or compensatory hyperextension of the back during overhead shoulder movement [17]. Although these tests were not performed in this case, clinicians should consider utilizing them when SANP is suspected.

While recovery from neuralgic amyotrophy is typically slow, the patient in this case demonstrated relatively rapid improvement in functional shoulder range of motion over the initial four treatment sessions, with intrasession changes of 10-20° following manual therapy interventions. Several mechanisms may have contributed to this improvement, including pain inhibition, increased trapezius and shoulder muscle activation, increased neuromuscular drive, and improved coordination of scapular upward rotation and posterior tilting [2,8,10,11,18-20]. The positive response to manual therapy in this case is consistent with previous studies identifying elastic therapeutic taping, dry needling, MWM, and thoracic manipulations as beneficial treatment techniques for neuralgic amyotrophy [2,10,12]. This rapid improvement in shoulder flexion range of motion highlights the potential role of manual therapy in the early management of this condition.

Importantly, the patient was able to maintain these improvements between sessions through performance of his home exercise program focusing on scapular stabilization exercises. Studies on neuralgic amyotrophy have recommended emphasizing scapular motor control, coordination, endurance, and self-management strategies but have not recommended early strengthening exercises [2,3,6,10]. The patient in this case responded well to a combination of scapular stabilization exercises and progressive shoulder strengthening, with improvements in strength in all assessed muscles except the lower trapezius. Continued weakness in the lower trapezius has been reported in other cases of SANP and may persist due to its distal innervation along the SAN [2,21].

Due to persistent trapezius weakness, the patient was unable to return to full-duty work at the conclusion of therapy. The final outcome measurements were mixed, with improvements on the GRC and PROMIS self-efficacy scales but decreased scores on the PROMIS physical function subscale. This decline on the PROMIS physical function subscale was not consistent with the patient’s reported subjective improvement and objective measurements. This discrepancy may reflect increased insight by the patient into his physical limitations or a change in perception of his functional abilities following the diagnosis of SAN injury.

## Conclusions

Physical therapists should be aware that neuralgic amyotrophy affecting the SAN is a rare cause of scapular dyskinesis. Clinicians should suspect SANP in patients who demonstrate compensatory scapular protraction during shoulder flexion with significant improvement in motion during the modified SAT, along with trapezius weakness and atrophy. Early conservative management with physical therapy using a multimodal approach, including a combination of manual therapy techniques, was associated with rapid restoration of shoulder flexion range of motion within the first four weeks of treatment in this case. In addition, the patient demonstrated improvements in shoulder strength and pain management following physical therapy intervention. These results indicate that physical therapy, including manual therapy, can be beneficial in the early conservative management of this condition.
